# Can monetary incentives overturn fairness-based decisions?

**DOI:** 10.1098/rsos.211983

**Published:** 2023-06-21

**Authors:** Martin Weiß, Anne Saulin, Vassil Iotzov, Johannes Hewig, Grit Hein

**Affiliations:** ^1^ University Hospital Würzburg, Center of Mental Health, Department of Psychiatry, Psychosomatics and Psychotherapy, Translational Social Neuroscience Unit, Margarete-Höppel-Platz 1, Würzburg, 97080, Germany; ^2^ University of Würzburg, Institute of Psychology, Department of Psychology I: Differential Psychology, Personality Psychology and Psychological Diagnostics, Marcusstr. 9–11, Würzburg, 97070, Germany

**Keywords:** fairness, financial incentives, punishment, compensation, drift-diffusion modelling

## Abstract

Fairness norms and resulting behaviours are an important prerequisite for cooperation in human societies. At the same time, financial incentives are commonly used to motivate social behaviours, yet it remains unclear how financial incentives affect fairness-based behaviours. Combining a decision paradigm from behavioural economics with hierarchical drift-diffusion modelling, we investigated the effect of different financial incentives on two types of fairness-based decisions in four experimental groups. In two groups, participants divided points between themselves and a disadvantaged person, inciting fairness-based compensation behaviour, in two other groups they divided points between themselves and a fairness violator, inciting fairness-based punishment behaviour. In addition, each group received financial incentives that were either aligned or in conflict with the respective fairness-based behaviour. This design allowed us to directly investigate how different incentives shape the cognitive mechanism of fairness-based decisions and whether these effects are comparable across different fairness domains (fairness-based punishment versus fairness-based compensation). Results showed that offering conflicting incentives diminished fairness-congruent decision behaviour and rendered the fairness-congruent decision process less efficient. These findings demonstrate that financial incentives can undermine fairness-based behaviour, and thus are relevant for the development of incentive schemes aimed at fostering cooperative behaviour.

## 1. Introduction

Fairness is one of the strongest social norms in social decision-making. Documenting the importance of fairness as one of the key norms guiding human behaviour [1], fairness-based behaviours have been the focus of experimental economist theories as well as research on social preferences (e.g. [2,3]). Following these theories, the strength of the fairness norm is derived from the equality of resource allocation [4]. Accordingly, individuals trade fairness concerns against their goal to increase material resources in order to pursue more equal outcomes [4,5]. An equal distribution of resources is perceived as fair, especially for unexpected gains (windfall money; *principle of equality*; [6]). In cases in which resources (i.e. money) are earned from a real effort task, fairness is rather associated with the *principle of equity*, i.e. a fair distribution of resources depends on the efforts of the stakeholders [7–10]. In many empirical studies, fairness is investigated using paradigms from behavioural economics such as the dictator game (where credits are allocated by only one person—the dictator or allocator; [11]) or the ultimatum game (where one person—the proposer—proposes a certain distribution of credits or money and the other person—recipient—can accept or decline the proposed share). Here, fairness is conceptualized as an equal sharing of resources, i.e. as an equality norm or ‘egalitarian fairness norm' [12]. This fairness norm is violated by an unequal distribution of resources, in particular self-advantageous inequality created by the norm violators [13,14].

Although participants on average do not choose the exact equal distribution (e.g. in the dictator game on average 70% for the dictator herself leaving 30% for the receiver; [15]), there is ample evidence that individuals react strongly when observing a violation of the egalitarian fairness norm (e.g. [16–20]). Often, observers of a norm violation can choose to either punish the norm violator [3,21–23] or to compensate the disadvantaged [24,25]. Both fairness-based punishment and fairness-based compensation can result in a deviation from the equality norm, i.e. the norm violator might end up with less than the disadvantaged and the disadvantaged might end up with more than the norm violator. Despite this inequality in the outcome, the act of punishment or compensation in response to the norm violation is perceived as fairness-based behaviour (and may even be understood as a situation-contingent norm itself; [26]).

In more detail, the option to punish observed unfairness has been investigated in so-called third-party punishment games [27]. Here, participants can invest their own credit to sanction a norm violation that affected other players [3,21–23]. The frequency and extent of fairness-based punishment behaviour such as third-party punishment are shaped by the cost associated with the decision to punish a norm violator [22,28–33] as well as individual differences in prosocial traits [34].

In recent years, an increasing number of studies have investigated the alternative option to react to an observed violation of the egalitarian fairness norm, namely the option to compensate for the individuals' disadvantage resulting from the norm violation (fairness-based compensation; [24,25]). There is evidence that individuals prefer to compensate the victim of a violated fairness norm even when punishment prevents future norm violations (e.g. [35]), and forgo more money to compensate others for unfair pain stimulation than to alleviate their own pain [36]. These results suggest that compensating a victim of unfair treatment is an important measure for achieving what is perceived as fair in this specific situation by overruling one's own profit.

Studies in which participants were able to choose between punishment and compensation have yielded mixed results with evidence in favour of preferring punishment as well as preferring compensation depending on the context [18,37–39]. Further, there are studies testing the consequences of compensation behaviour as well as punishment behaviour to establish fairness for the third-party decision-maker [22,40]. For instance, Raihani & Bshary [40] investigated how uninvolved bystanders punish or reward third-parties who have punished unfair allocators, compensated unfairly treated receivers or did not act at all. The results showed that bystanders rewarded third-parties more for acting as opposed to doing nothing. Additionally, bystanders were more likely to reward another player for third-party compensatory behaviour towards the receiver than for third-party punishment of the unfair allocator. In line with these results, there is evidence that participants preferred individuals who compensated others compared with individuals who punished others across a variety of economic paradigms [41]. This evidence indicates that compensatory action may be more socially accepted than punitive action.

In sum, these previous studies indicate that restoring perceived fairness by punishment or compensations is based on cost considerations for both types of behaviour. However, challenging this view, there is also evidence showing that individuals maximize their own outcome instead of choosing an equal distribution or compensating the victims of previous unfair behaviour (e.g. [42–44]). These results suggest that reactions to a violation of the egalitarian fairness norm (such as fairness-based punishment or fairness-based compensation) can be undermined by the selfish motive of outcome maximization (e.g. the opportunity to gain additional financial reward). However, so far it remains unclear how financial incentives (inducing a selfish motive) affect the processing of decisions that result in fairness-based punishment or compensation.

**Figure 1.**
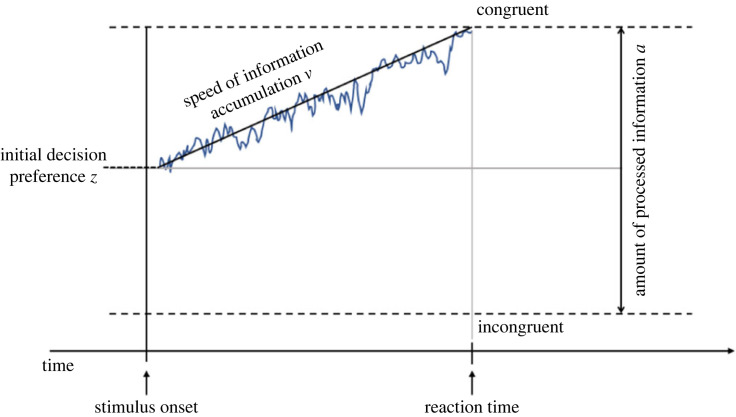
Schematic illustration of the DDM. The DDM conceptualizes the decision-making process as a noisy accumulation of information (blue line). This process can be characterized in terms of the speed of information uptake (*v-*parameter), the *initial bias* towards one of the decision options (*z-*parameter), and the amount of information required until a decision is reached (*a* parameter). Please note that values of the drift rate above the zero point of the accumulation process are positive, i.e. with a drift towards the upper boundary (here, the fairness-congruent option, i.e. decisions in favour of the other in the compensation groups and decisions in favour of the self in the punishment groups), and values below zero are negative [47], i.e. with a drift towards the lower boundary (here, the fairness-incongruent option, i.e. decisions in favour of the self in the compensation groups and decisions in favour of the other in the punishment groups).

One promising approach to investigate the effect of one variable (here financial incentives) on specific subcomponents of the decision process is drift-diffusion modelling (DDM). Drift-diffusion models use the combined information of participants' reaction times and choices to characterize how noisy information is accumulated to select a choice option (here the decision to restore fairness via compensation or via punishment) based on three different parameters (the *v-*, *z-* and *a-*parameters; [45,46]; figure 1). The *v-*parameter (drift rate) describes the speed at which information is accumulated to choose one of the options, i.e. the efficiency of the decision process itself. The *z-*parameter (initial bias) reflects the initial decision preference, i.e. the extent to which an individual prefers one of the decision options before making the decision. The third component, the *a-*parameter, quantifies the amount of information required to choose one of the options. The higher the value the more careful (conservative) the decisional style.

Recent studies using DDMs to model social decision processes have shown that the *v-*parameter, i.e. the efficiency of the decision process (drift rate), can be influenced by whether a potential reward linked to task performance will be paid out to themselves or another person [48], or whether the information offered for choosing a certain option is linked to a desirable or an undesirable reward structure [49]. The *z*-parameter (initial bias) was also found to be affected by different types of reward and motivation-related factor. Chen & Krajbich [50], for example, showed that participants with higher baseline prosociality exhibit a larger initial bias towards making prosocial choices. Likewise, the combination of social motives [51], a single peer's decision behaviour [52] as well as social conformity, i.e. group decision behaviour [53] can increase the bias towards the respective congruent choice option. The role during norm violation, i.e. whether participants were in the role of the observer or the victim altered both the *v-* and the *z*-parameter of the decision process [54]. Changes in the decision threshold were less frequently reported (but see [54]).

Here we use DDM to investigate to what extent the cognitive mechanism of fairness-based decisions changes if fairness is challenged or enhanced by a selfish motive, and whether these effects are comparable in fairness-based punishment and fairness-based compensation.

To do so, we designed a paradigm in which the selfish motive of outcome maximization (i.e. a monetary incentive for a certain behaviour, cf. [55–57]) is in conflict or aligned with either fairness-based punishment or with fairness-based compensation behaviour (table 1). Participants were assigned to one of four groups. In all groups, participants divided points between themselves and two targets, with the target varying by trial. In all groups, one of the targets was a previously uninvolved player (‘baseline’). The other target was either the allocator or the recipient in-game the participant previously observed. This resulted in four groups according to a two-by-two crossover design with the factors of fairness domain (punishment versus compensation) and conflict degree (conflicting versus aligned). Based on this design, we could directly compare how fairness-based punishment and fairness-based compensation decision processes were altered by incentives that potentially enhance or challenge the respective fairness norms.

**Table 1.** Overview of the manipulation dimensions (incentive and fairness domain) resulting in four groups according to the manipulation dimensions fairness domain (fairness-based punishment versus fairness-based compensation) and incentive (for decisions in favour of the other or decisions in favour of the self). The type of incentive is either aligned with fairness-based behaviour or in conflict with fairness-based behaviour.

incentive	fairness domain	
	fairness-based punishment	fairness-based compensation
decisions in favour of the other incentivized	conflict punishment group	aligned compensation group
decisions in favour of the self-incentivized	aligned punishment group	conflict compensation group

**Figure 2.**
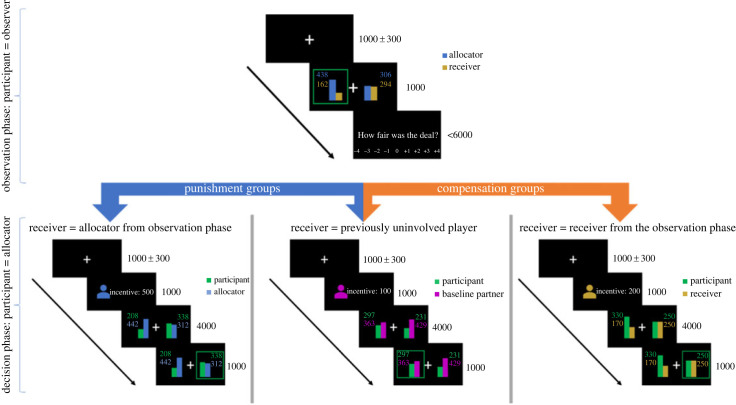
The trial procedure of the observation phase (top), example trials of the decision task for all groups (bottom), and the different experimental conditions (types of the receiver) in the punishment groups (blue arrow) and the compensation groups (orange arrow). Each trial of the observation phase (top) started with a fixation period. Then, participants observed allocation decisions in a binary dictator game, most of which were unfair (80% unfair, 20% fair). In the example trial shown here, the allocator assigned 102 points to the receiver and 498 points to the self. The decision of the allocator was highlighted by a green rectangle. After having observed the decision of the allocator, participants were asked to rate the fairness of the decisions on a 9-point scale ranging from very unfair (−4) to very fair (+4). In the decision task (bottom), participants were the allocators. In the two punishment groups, they divided points between themselves and the allocator of the previous observation phase (left panel) or to self and a previously uninvolved player (baseline partner; middle panel). In the two compensation groups, participants divided points between themselves and the receiver of the previous observation phase (right panel) or to the self and a previously uninvolved player (baseline partner; middle panel). The type of receiver (allocator of the observation phase versus uninvolved player; receiver of the observation phase versus uninvolved player) was indicated by a cue following the fixation period. In parallel, the incentive was shown (varying between 0 and 500 points). Depending on the experimental condition (table 1), the incentive was provided for decisions in favour of the self or in favour of the other. Next, participants were presented with the decision options, and allocated points in favour of the self or in favour of the respective other (allocator/receiver of the observation phase; uninvolved player). The decision was highlighted by a green rectangle.

The first group of participants divided points between themselves and a previously unfair allocator (i.e. a person who should be punished to restore the fairness norm, figure 2, bottom left panel) as well as themselves and a previously uninvolved player (baseline partner), and additionally receives financial incentives for decisions in favour of the other (figure 2, bottom middle panel). Incentives for decisions in favour of the other were in conflict with the fairness-based punishment behaviour (i.e. the fairness-incongruent choice option) because they reward the decision that maximizes the outcome of the allocator instead of punishing to restore fairness (*conflict punishment group*). A second group of participants again divided points between themselves and the allocator and a previously uninvolved player (baseline partner) but received financial incentives for decisions in favour of the self. Incentives for decisions in favour of the self were aligned with fairness-based punishment (i.e. the fairness-congruent choice option) because they rewarded the decisions that minimized the outcome of the allocator (*aligned punishment group*). A third group of participants divided points between themselves and a previously unfairly treated receiver (receiver, i.e. a person that should be compensated to restore the fairness norm, figure 2, bottom left panel) as well as a previously uninvolved player (baseline partner, figure 2, bottom middle panel), and additionally received a financial incentive for decisions in favour of the other (i.e. the fairness-congruent choice option). This incentive was aligned with fairness-based compensation because it rewarded the decisions that maximized the outcome of the receiver (*aligned compensation group*). A fourth group of participants again divided points between themselves and the receiver and a previously uninvolved partner (baseline partner) but received incentives for decisions in favour of the self (i.e. the fairness-incongruent choice option). These were in conflict with fairness-based compensation because they rewarded the decisions that minimized the outcome of the receiver instead of compensating this person (*conflict compensation group*).

### 1.1. Manipulation check

As a prerequisite for interpreting the results, we assumed that the fairness norm increases the likelihood to decide in favour of the self towards the allocator (fairness-based punishment) and increases the likelihood to decide in favour of the other towards the receiver (fairness-based compensation) as compared with the respective baseline partner without previous fairness association (hence, aligned incentives increase the frequency of fairness-congruent responses). We further assumed that higher incentives to choose in favour of the self should increase the frequency of decisions in favour of the self, and higher incentives to choose in favour of the other should increase the frequency of decisions in favour of the other.

### 1.2. Hypotheses

The hypotheses refer to the trials with allocations towards the allocator/receiver. For the two conflict groups (*conflict punishment group, conflict compensation group),* we hypothesized less frequent decisions in favour of the self in the *conflict punishment group* and less frequent decisions in favour of the other in the *conflict compensation group* as compared with the respective aligned groups (*aligned punishment group, aligned compensation group*) (H1). This effect should be stronger, the larger the respective trial-wise incentive (H2). Further, we hypothesized that the effect of conflict on the participants' decisions would be different in the fairness-driven punishment domain compared with the compensation domain. Accordingly, the *conflict punishment group* versus *aligned punishment group* contrast (punishment domain) should be different from the *conflict compensation group* versus *aligned compensation group* contrast (compensation domain; H3).

According to the DDM, an incentive-induced conflict (i.e. effects in line with H1) may be reflected by (i) a reduced speed of information accumulation towards the congruent option (H4) (i.e. reduced *v-*parameter towards the congruent decision option due to a reduced motive to punish or to compensate due to the conflicting incentive, cf. [49]), (ii) a shift of the starting point away from the congruent decision option (H5) (i.e. decreased *z-*parameter with respect to the congruent decision option due to a reduced motive to punish or to compensate due to the conflicting incentive, cf. [51]), or (iii) the assumed alterations in both parameters.

Further, we hypothesized that differential effects of conflict degree in the different fairness domains may be reflected by different degrees of influence on the, respectively, altered parameter(s) (H6).

## 2. Material and methods

### 2.1. Power analysis

To estimate the sample size, we used the ‘simstudy’ package [58] in Rstudio [59] to simulate data and ‘simR' [60] to compute power calculations. The simulations (code openly available at https://osf.io/bgka8) were based on our hypothesized effects informed by previous studies [51,61–63]. We thus set the expected probabilities for decisions in favour of the other at 65% in the two compensation groups and at 45% in the two punishment groups. In accordance with a larger effect of conflicting incentives in the punishment domain, we weighted the effect of incentive level on participants' decision behaviour with 0.25 in the *conflict punishment group* and 0.15 in all other groups. The model on which the estimations were based was specified as follows:

response∼conflict_degree ∗ fairness_domain+conflict_degree ∗ incentive_level+(1|id).

Thus, participants' responses (congruent option versus incongruent option) were predicted by the fixed effects conflict degree, fairness domain, incentive level, as well as the interaction of conflict degree and fairness domain and conflict degree and incentive level. Participants were included as a random intercept.

With *N* = 30 per group, i.e. 120 participants in total, the achieved power for the interaction between the fairness domain and conflict degree was 95% (95% CI [88.72, 98.36]) and greater than 99% (95% CI [96.38, 100]) for the interaction between conflict degree and incentive level; for details of the models, see ‘Confirmatory analyses'. We continued recruitment until we obtained complete data from 120 participants (i.e. 30 per group) who passed all attention checks (see ‘Data analysis').

### 2.2. Participant details

All participants were native German speakers, in the age range between 18 and 45 years. All participants gave informed consent. The study was approved by the Ethics Committee of the Faculty of Psychology of the University of Würzburg (GZEK 2021-47). Participants received monetary compensation of 3€, plus payout from two randomly chosen trials of the decision task and a possible incentive. In sum, they received between 3.70 and 13.20€ depending on their decisions (for details, see ‘Decision task’). The whole experiment took approximately 30 min.

### 2.3. Experimental procedure

To assess the effect of the fairness norm and its interaction with financial incentives in a large and diverse sample (there is evidence for great variety in third-party punishment for unfair behaviour at least in the ultimatum game across different populations [64]), we developed an online version of the task and collected data for the different groups via an online platform (www.clickworker.com). Our own previous studies and the studies of others [65] have verified the quality and temporal accuracy of data acquired in online paradigms on this platform.

For a clear interpretation of potential effects in the decision phase, it is important that all participants observe the same decision pattern (80% unfair decisions) in the observation phase. To achieve that, participants were presented with a selection of binary dictator game decisions that were collected in our laboratory before the online study (see e.g. [66,67] for a comparable approach). The payout resulting from this study was transferred to the laboratory participants. The payout resulting from the receiver and the baseline partner were transferred to other online participants. Participants were informed that they would observe a selection of decisions of individuals who played the game before in the laboratory and who received the payout of two randomly selected trials. Participants were informed immediately after the decision task, that the selection of the observed decisions was biased such that all participants observed the same frequency of decisions in favour of the self.

**Figure 3.**
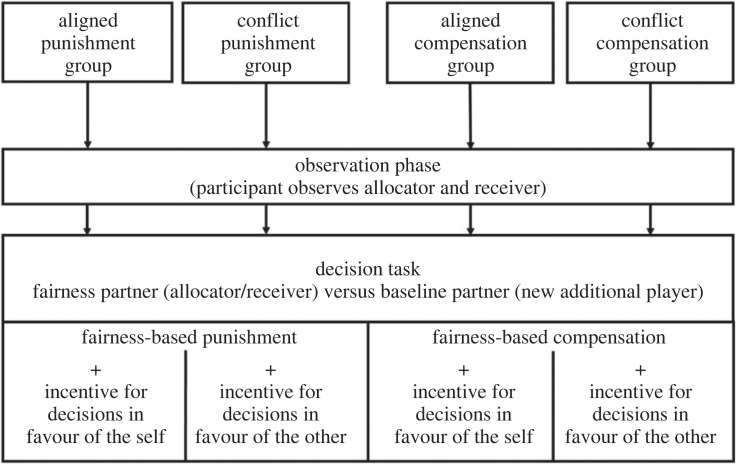
Exemplary experimental procedure. Each participant was assigned to one of four groups. During the observation phase, in which the participant was merely in the observer role, the fairness motive was associated with two other players (the allocator or the receiver). In the following decision task, participants were in the role of the allocator and divided points between themselves and the previously observed fairness partner (allocator or receiver) as well as themselves and a baseline partner (previously uninvolved player). Depending on the group assignment, participants were offered trial-by-trial varying incentives for making decisions in favour of the self, or for making decisions in favour of the other. For details regarding each task, please refer to figure 2.

In each of the four groups, the study consisted of an interaction partner observation phase (figures 2 and 3), followed by the decision task. During the observation phase, participants observed how one person (allocator) made decisions in favour of the self at the cost of another person (receiver). After the observation phase, all participants performed the identical decision task in which they could allocate points in favour of themselves or in favour of other players. In a two × two design (table 1), half of the participants divided points between themselves and the previously observed allocator (*conflict punishment group* and *aligned punishment group*) and the other half made the same decisions towards the previously observed receiver (*conflict compensation group* and *aligned compensation group*). According to conflict degree, participants in the *aligned punishment group* and the *conflict compensation group* were incentivized for making decisions in favour of the self, while the *conflict punishment group* and the *aligned compensation group* were incentivized for making decisions in favour of the other. Incentives were thus either in conflict or aligned with the respective fairness-driven behaviour. In all groups, participants made the same decisions towards the respective fairness partner as well as towards a new player they had not observed before (baseline partner).

Participants and the other players were assigned their different roles in each of the phases (observation phase and decision phase) by a manipulated assignment. In the observation phase, the participant was assigned the observer role while the other two ostensible players were assigned the decider role. In the decision task, the participant was assigned the decider role.

After completing the tasks, participants were asked to fill out the trait items of the German version of the state-trait-anger-expression-inventory (STAXI; [68,69]), the justice sensitivity short scales [70] and the subscale altruism of the revised version of the prosocial tendencies measure [71,72].

### 2.4. Reward structure

**Figure 4.**
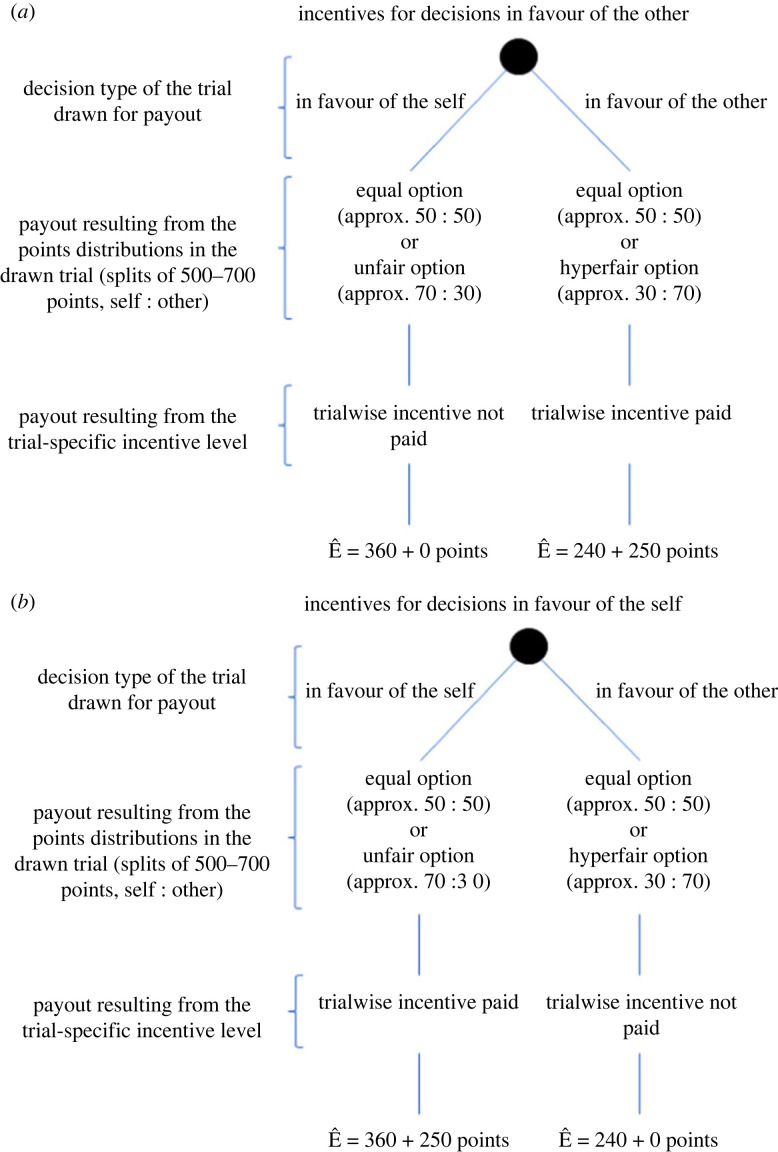
Overview of the reward structure. (*a*) In the two groups, in which decisions in favour of the other are incentivized (conflict punishment group, aligned compensation group), participants received their pay out based on the points of the chosen distribution. Additionally, they received the pay out based on the trial-specific incentive for the drawn trial(s) in which they chose in favour of the other. (*b*) In the two groups, in which decisions in favour of the self were incentivized (aligned punishment group, conflict compensation group), participants received their pay out based on the points of the chosen distribution. Additionally, they received the pay out based on the trial-specific incentive for the drawn trial(s) in which they chose in favour of the self.

Participants' trial-by-trial reward and corresponding payout were influenced by the trial-wise incentive level as well as the respective point distributions (figure 4).

### 2.5. Incentive levels

The incentive level varied trial-by-trial. It comprised six levels ranging from 0 to 500 points in steps of 100, with 10 trials per level.

### 2.6. Distributions

In order to control for the effects of inequality aversion, which describes people's tendency to prefer equal allocations and avoid unequal allocations [4], equal and unequal point distributions equally often constituted the option in favour of the self and in favour of the other. That is, the *in favour of the self option* could either be an unfair point distribution (more points for the participant) or an equal distribution (approximately equal amount of points for the participant and the respective partner). The *in favour of the other option* could be either equal or a hyperfair point distribution (more points for the respective partner). Therefore, decisions in favour of the other and in favour of the self in our task were always relative to the two options that were presented in the respective trial.

These options were created based on different distributions of splits of 500–700 points in steps of 10 points. The unfair distributions yielded split ratios of 70 : 30 (self : other) with a jitter of 5% (e.g. the participant would receive 449 points and the partner 201 points when splitting 650 points). The hyperfair distributions yielded the opposite split ratio of 30 : 70 with a jitter of 5% (i.e. this would for example entail a gain of 192 points for the participant and 488 points for the partner when splitting 680 points). The equal distribution referred to distributed points at a ratio of approximately 50 : 50 (e.g. 283 points for the self and 277 points for the other when splitting 560 points). We used a range of ±5% to avoid a decline in attention to task, which might occur if options were exact multiples of 10 across all trials or if participants would face exactly equal options in the equal distributions.

In each trial, an equal distribution of points was simultaneously shown with either an unfair or a hyperfair distribution. As a result, the *in favour of the other option* could either correspond to the hyperfair distribution (in trials showing a hyperfair and an equal distribution) or to the equal distribution (in trials showing an equal and an unfair distribution). The *in favour of the self option* could correspond to the unfair distribution (in trials showing an unfair and equal distribution), but also to the equal distribution (in trials showing an equal and a hyperfair distribution). This experimental set-up allowed us to disentangle the effect of the preference for more equal decision options and the preference for a decision in favour of the self versus in favour of the other.

Each incentive level (0–500 points, i.e. six levels) was paired 10 times with each of the two possible combinations of distributions (i.e. hyperfair versus equal and unfair versus equal), resulting in 10 (number of repetitions) × 2 (distribution combinations) × 6 (incentive levels) = 120 trials per interaction partner.

### 2.7. Decision task

The decision task (figure 2, bottom panels) was a modified version of a two-alternative-forced-choice adaptation of the commonly used dictator game [11], which has been successfully used in previous studies [50,73]. The task was identical in all groups. In each trial, participants divided the money between themselves and their respective partner. Participants could choose between two distribution options, one of which was in favour of the other and one in favour of the self.

After an initial fixation cross (1000 ± 300 ms), an icon cue indicated the person who was affected by the subsequent decision (allocator or receiver or baseline partner), and at the same time, the amount of the incentive associated with the current trial was displayed next to the icon (1000 ms). Afterwards, the allocation options were presented using different colours for the different players, and the participants made their decision by pressing the left or right arrow key within 4000 ms. The position of the two allocation options was randomized across trials to minimize response biases due to motor habituation. After the participants had responded, but at the earliest after 1000 ms (to avoid careless responding), a green box appeared for 1000 ms around the selected allocation. The decision task consisted of 120 decisions towards the allocator/receiver and 120 decisions towards the baseline partner in random order (i.e. 240 trials in total).

Before starting the experiment, participants conducted 12 practice trials (six for the observation phase and six for the decision task). Additionally, we randomly placed six control trials as a means to ensure data quality (i.e. to detect careless responding, see below). These control trials were constructed such that the same option maximizes the participant's and the partner's points. Thus, not choosing the mutually beneficial option that is most profitable for both players would indicate sloppy responding.

Together, the decision task consisted of 240 experimental trials (i.e. 2 partners, 6 levels of incentive, 2 pairings of decision option distributions and 10 repetitions of each trial), 20 observation trials, 12 practice trials and 6 control trials, resulting in a total of 278 trials. In the case of missed trials (i.e. response time greater than 4000 ms), a repeat loop ensured that the participants reached a total of 120 trials per partner.

### 2.8. Observation phase

In the observation phase, participants observed the repeated decisions made by an unfairly behaving allocator (allocator) towards the unfairly treated receiver (receiver). Each observation trial started with a jittered (1000 ± 300 ms) fixation cross followed by the two options for the ostensible allocator (in favour of the self and in favour of the other). The option selected by the allocator (green rectangle) was displayed for 1000 ms (figure 2, top panel). The following rating scale was shown for a maximum of 6 s. Participants evaluated the fairness of the decision (‘How fair was the deal?' in German). The scale ranged from −4 (*very unfair*) to +4 (*very fair*) and was visually displayed in steps of 1. The fairness ratings were added to ensure that participants paid attention to the distribution of credits during the observation phase, which was crucial for implementing our experimental conditions. The observation phase contained 20 trials, with 16 decisions in favour of the self (80%) and 4 decisions in favour of the other (20%) made by the allocator. Analogously to the distributions in the decision task, 10 of these trials had the distribution pairing hyperfair versus equal with 8 being decided in favour of the self (allocator) and 2 being decided in favour of the other (receiver). The other 10 trials had the distribution pairing unfair versus equal with 8 being decided in favour of the self (allocator) and 2 being decided in favour of the other (receiver).

### 2.9. Payout

Participants received a show-up fee of 3€ for participating in the experiment. In order to ensure that participants regarded all trials as equally relevant for additional payout, they were told that two of the decisions they made (after the practice trials) would be randomly selected and would serve as additional payout resulting in an additional 0.70–2.60€ per trial, with 100 points corresponding to 0.50€ rounded to the next 0.10€ amount, depending on the trials drawn.

Likewise, participants were told that they would receive the additional payout associated with the incentive level in the trials drawn if their decision corresponded to the incentive criterion (i.e. they received the incentive of that trial if they made the decision in favour of the other in the *aligned compensation group* and the *conflict punishment group* and if they made the decision in favour of the self in the *conflict compensation group* and the *aligned punishment group*). Here too, 100 points corresponded to 0.50€, resulting in an incentive-based pay-out of an additional 0–2.50€.

Hence, overall, participants received 3.70–13.20€ for participation, depending on the trials drawn and the participants' decision behaviour.

A participant's payout in group *g* based on drawn trial *n* payout*_gn_* can be described in terms of the following equation:

$${\mathrm{payout}}_{gn}=\{\begin{array}{cc} \{{\mathrm{points}}_{\mathrm{self}}(d_{gn})+\mathrm{incentive}(d_{gn})\}\;*\;0.005, & \mathrm{if}\;T(d_{gn})=I_{g}\;\;\\ \{{\mathrm{points}}_{\mathrm{self}}(d_{gn})\}\;*\;0.005,\;\; & \mathrm{if}\;T(d_{gn})\neq I_{g} \end{array},$$

with *d_gn_* = chosen distribution in drawn trial *n* for participants in group *g*, *T*(*d*_gn_) the decision type of the drawn trial *n* in group *g* (i.e. in favour of the self or in favour of the other) and *I_g_* the group-specific incentivized decision type (i.e. in favour of the self in the aligned punishment and conflict compensation groups and in favour of the other in the conflict punishment and the aligned compensation groups).

### 2.10. Data preparation

We excluded the entire datasets of participants who have failed to choose the mutually more profitable option in at least two of the six control trials. This exclusion criterion was implemented in the online experiment, such that we only stored datasets that met this criterion. Participants with an average response time of less than 500 ms were also excluded, as it could not be ensured that they completed the task carefully. In the 120 valid datasets obtained from clickworker, no participant had to be excluded based on this second criterion.

Participants' decisions in favour of the other versus *in favour of the self option* were recorded in order to correspond to our framework of fairness-driven behaviour and enable comparison across the two fairness domains. Therefore, we computed the variable ‘response' (congruent option chosen versus incongruent option chosen) based on the combination of the fairness domain (punishment versus compensation) and incentive (in favour of the other versus in favour of the self). Thus, a value of 1 means that the participant chose the respective congruent option (i.e. the *in favour of the other option* in the compensation groups and the *in favour of the self option* in the punishment groups) and a value of 0 reflects the participants' decision of the respective incongruent option (i.e. the *in favour of the other option* in the two punishment groups and the *in favour of the self option* in the two compensation groups).

### 2.11. Data analysis

Data curation and analyses were conducted in RStudio [59] using the lme4 package [74]. DDM was conducted using the python implementation of HDDM [75].

### 2.12. Details of the drift-diffusion modelling

The parameters for DDM were derived from the participants' decisions and reaction times. We applied hierarchical drift-diffusion modelling (HDDM), which is a version of the classical DDM that exploits between-subject and within-subject variability using Bayesian parameter estimation methods [76]. Hierarchical DDM already yields robust results at trial numbers around 40 [75] and reaches the plateau of near-optimal parameter estimation between 80 and 100 trials. Including 120 trials in the present paradigm should hence allow for robust and reliable parameter estimation. Based on the power analysis, a true choice effect should be detected with a 99% probability. Since DDM combines the information of reaction time and choices, it is more sensitive to potential effects than mere choice data alone (e.g. [46]). Reliable estimation of the underlying choice parameters should hence uncover the potential effects of monetary incentives on the fairness-based punishment and fairness-based compensation decision process.

We followed the recommendation of other studies using paradigms with identical experimental setting across conditions and estimated one *t_0_* parameter across all conditions [77–79]. This parameter indicates the duration of all extra-decisional processes such as basic encoding or motor processes [80]. Likewise, since our hypotheses focused on the *v*-parameter and the *z*-parameter, the *a*-parameter was fixed across all conditions.

The models were compared using the deviance information criterion (DIC; [81]). More recently, the Watanabe Akaike/widely applicable information criterion (WAIC) has been used more frequently (e.g. [82,83]). However, DIC and WAIC appear to practically show high consistency [84]. We hence focused on the DIC for model comparison. If the DIC of the more complex model was 10 higher than the comparison model, the more complex model was selected [85]. If not, model averaging was used in order to obtain the relevant parameter traces for hypothesis testing.

Model convergence was checked by visual inspection of the estimation chain of the posteriors, as well as by computing the Gelman–Rubin Geweke statistic for convergence (all values less than 1.01; [86]). Hypotheses were tested by comparing the traces of the relevant parameters based on the winning model(s). A difference was assumed to be ‘significant' if the probability for the two traces to not overlap was higher than 95% [75].

Based on previous studies employing a comparable task [51,63,73], participants have a slight bias towards making decisions in favour of the other. This bias, however, was smaller in online participants [63]. That is, in the online setting, participants chose the option in favour of the other in about 65% of the trials. Offering an additional incentive for making decisions in favour of the other increased the probability of making a decision in favour of the other by 12% in a laboratory study [62]. Given these results, a floor or ceiling effect based on the additional incentive should be unlikely in this study.

In this study, we also activated the fairness norm either promoting fairness-based punishment or fairness-based compensation. In order to test this unknown effect, we included a baseline partner towards whom the fairness norm was not experimentally activated. Taken together, previous results allowed for an estimation of neutral behaviour as well as incentive-driven behaviour on a group level and the baseline partner allowed for an estimation of the effect of the respective fairness-based behaviour on a subject level. With respect to the estimation of the DDM parameters, even the condition with the largest number of congruent responses should not pose a problem, since model estimation is robust even for over 90% accuracy [47].

### 2.13. Confirmatory analyses

#### 2.13.1. Manipulation check

In order to test for the main effects of fairness motive and conflict degree, for each fairness domain we conducted one logistic mixed models analysis with interaction partner (allocator/receiver versus baseline) and conflict degree (conflict versus aligned) as fixed effects and distribution type (four levels: 50 : 50 versus 90 : 10, 50 : 50 versus 70 : 30, 50 : 50 versus 30 : 70, 50 : 50 versus 10 : 90) as random slope, participant as random intercept and participants' responses (congruent versus incongruent) as a binary-dependent variable.

#### 2.13.2. Hypothesis testing

All analyses were performed only for the trials with the allocator and receiver as targets. According to our hypotheses for the fairness-driven punishment domain, we expected a main effect of conflict degree with a decreased number of congruent decisions in the *conflict punishment group* and the *conflict compensation group* as compared with the *aligned punishment group* and the *aligned compensation group* (H1). This effect should be stronger for larger incentives (H2). Further, we hypothesized that the effect of conflict on the participants' decisions would be stronger in the fairness-driven punishment domain than in the compensation domain (H3). In order to test these hypotheses, we conducted a logistic mixed model analysis with the factors conflict degree (conflict versus aligned), fairness domain (punishment versus compensation) and incentive level (six levels: 0, 100, 200, 300, 400, 500), and the two-way interactions between incentive level × conflict degree and conflict degree × fairness domain as fixed effects, participant as random intercept and participants' responses (congruent versus incongruent) as a binary-dependent variable. H1 would be confirmed if we observed a significant main effect of conflict degree and H2 would be confirmed if we observed a significant conflict degree × incentive level interaction. H3 would be confirmed if we observed a significant interaction between conflict degree and fairness domain.

In the next step, we tested whether the conflict between the fairness motive and outcome maximization differentially influenced components of the congruent versus incongruent decision processes in the two different fairness domains. To this end, we compared seven different DDMs.

Specifically, we compared the null model (M0, all parameters fixed across all conditions) with two sets of hierarchical DDMs (table 2 for overview). The first set of models assumed the *v*-parameter to vary by conflict degree (V1) or by conflict degree and fairness domain (V2). The second set of models assumed the *z*-parameter to vary by conflict degree (Z1) or by conflict degree and fairness domain (Z2). H4 (conflict modulates *v*-parameter) would be confirmed if one of the V models was among the winning models according to the DIC, and *v*_congruent_ was greater than *v*_incongruent_ (i.e. (*P*(*v*_congruent_) > *P*(*v*_incongruent_)) > 95%). H5 (conflict modulates *z*-parameter) would be confirmed if at least one of the *Z* models was among the winning models, and *z*_congruent_ was greater than *z*_incongruent_ (i.e. (*P*(*z*_congruent_) > *P*(*z*_incongruent_)) > 95%). H6 (conflict modulates *v*-parameter and *z*-parameter) would be confirmed if both criteria were met. Finally, H7 (the two fairness domains are differentially affected) would be confirmed if either model V2 and/or model Z2 was the winning model and P((*v*_aligned punishment_) − (*v*_conflict punishment_)) − ((*v*_aligned compensation_) − (*v*_conflict compensation_)) > 95% and/or P((*z*_aligned punishment_) − (*z*_conflict punishment_)) − ((*z*_aligned compensation_) − (*z*_conflict compensation_)) > 95% or vice versa (table 3).

**Table 2.** Study-design table.

question	hypothesis	sampling plan	analysis plan	rationale for deciding the sensitivity of the test for (dis-)confirming the hypothesis	interpretation given different outcomes
Is restorative fairness behaviour diminished when a conflicting monetary incentive is offered compared with when a non-conflicting (aligned) incentive is offered?	H1: Conflicting monetary incentives diminish fairness-based behaviour.	Simulation of data based on plausible effect sizes using *simr, resulting in N* *=* *120 participants.*	Logistic mixed model response (congruent versus incongruent)∼conflict degree (conflicting versus aligned) × fairness domain (punishment versus compensation) + conflict degree × incentive level (0–500) + (1 | participant)	Based on previous studies using a similar design, we simulated data using plausible outcome probabilities for the different conditions. The results of this simulation were used as a proxy of expected effect size and served as a basis for power estimations (see section *Power analysis* for details).	*significant main effect of conflict degree:*
					A. a smaller number of congruent responses in the conflict groups than in the aligned groups This result would confirm H1.
					B. a larger number of congruent responses in the conflict groups than in the aligned groups This result would contradict H1.
Is the effect of monetary incentive scaled by the size of the incentive?	H2: The larger, the incentive, the larger its effect.				*Significant interaction between conflict degree and incentive level:*
					A. the higher the incentive, the larger the effect of conflict degree: people scale the influence of the incentive according to the size of the conflictual incentive. This result would confirm H2.
					B. the higher the incentive, the smaller the effect of conflict degree: people scale the influence of the incentive inversely to the size of the conflictual incentive. This result would contradict H2.
Is fairness-based restorative justice behaviour equally influenced by fairness-based punishment and fairness-based compensation?	H3: Fairness-based punishment behaviour is differently influenced by conflicting as opposed to aligned monetary incentives as compared with fairness-based compensation.				*Significant interaction between the fairness domain and conflict* degree:
					A. larger effect of conflict degree in the punishment domain: fairness-based punishment behaviour is more easily influenced by the selfish motive of outcome maximization. This result would confirm H3.
					B. larger effect of conflict degree in the compensation domain: fairness-based compensation is more easily influenced by the selfish motive of outcome maximization. This result would confirm H3.
					*no significant interaction between fairness domain and conflict degree:*
					this result would contradict H3.
Which components of the punishment-based or compensation-based fairness decision process are influenced by an additional conflicting monetary incentive?	H4: Conflicting monetary incentives reduce the efficiency of the decision process for choosing the respective congruent decision option, reflected by a reduction of the *v*-parameter in the conflict groups compared with the aligned groups		Using hierarchical drift diffusion modelling (HDDM), we will compare which model best fits the data by comparing the WAIC (widely applied information criterion/Watanabe–Akaike information criterion) within our model space (for an overview of all models, refer to table 3).		models V1–V2 are among the winning models and *v*_congruent_ is greater than *v*_incongruent_ (i.e. (*P*(*v*_congruent_) > *P*(*v*_incongruent_)) >95%)
	H5: conflicting monetary incentives reduce the initial bias towards the respective congruent option, reflected by a reduced *z*-parameter in the conflict groups compared with the aligned groups				models Z1–Z2 are among the winning models and *z*_congruent_ is greater than *z*_incongruent_ (i.e. (*P*(*z*_congruent_) > *P*(*z*_incongruent_)) > 95%)
Are the respective components of the decision process equally influenced by fairness-based punishment and fairness-based compensation?	H6: Conflicting monetary incentives influence the respective component(s) of the decision process differently in fairness-based punishment than in fairness-based compensation.				Models V2 and/or Z2 are among the winning models and P((*v*_congruent punishment_) – (*v*_incongruent punishment_)) – ((*v*_congruent compensation_) – (*v*_incongruent compensation_)) > 95% and/or P((*z*_congruent punishment_) – (*z*_incongruent punishment_)) – ((*z*_congruent compensation_) – (*z*_incongruent compensation_)) > 95%

**Table 3.** Set of hierarchical DDMs: A null model (B0, all parameters fixed across all factor levels) was compared together with three models allowing the *v*-parameter to vary by conflict degree (V1) or by conflict degree and fairness domain (V2). It was also compared together with two models allowing the *z*-parameter to vary by conflict degree (Z1) or conflict degree and fairness domain (Z2).

	models	specification
null model	M0	—
influence on *v*-parameter models	V1	*v*∼*conflict degree*
	V2	*v*∼*conflict degree* *+* *fairness domain*
influence on *z*-parameter models	Z1	*z*∼*conflict degree*
	Z2	*z*∼*conflict degree* *+* *fairness domain*

## 3. Results

### 3.1. Manipulation check

To test the differential effect of monetary incentives on response behaviour, we conducted a logistic mixed model with response (congruent versus incongruent) as a dependent variable and interaction partner (baseline versus fairness partner), conflict degree (aligned versus conflict) as predictors. Interaction partner × conflict degree was included as fixed effects, participant as a random intercept, and distribution type as random slope (four levels: 50 : 50 versus 90 : 10, 50 : 50 versus 70 : 30, 50 : 50 versus 30 : 70, 50 : 50 versus 10 : 90). For the punishment domain, results showed a tendency for more aligned responses towards the fairness partner than the baseline partner (*β* = 0.16, s.e. = 0.09, *z* = 1.75, *p* = 0.08), significantly less aligned responses in the conflict groups than in the aligned groups (*β* = −1.55, s.e. = 0.56, *z* = −2.77, *p* = 0.006) and a trend for an interaction effect (*β* = −0.23, s.e. = 0.12, *z* = −1.95, *p* = 0.05). For the compensation domain, results also showed a tendency for more aligned responses towards the fairness partner than the baseline partner (*β* = 0.15, s.e. = 0.08, *z* = 1.82, *p* = 0.07), significantly less aligned responses in the conflict groups than in the aligned groups (*β* = −3.57, s.e. = 0.70, *z* = −5.08, *p* < 0.001) and an interaction effect (*β* = 0.31, s.e. = 0.13, *z* = 2.33, *p* = 0.02). The results confirm that the monetary incentives affected participants' responses, but do not test the successful implementation of the fairness manipulation. To rectify this, we added a logistic mixed model analysis with the binary-dependent variable decision (in favour of the other versus in favour of the self), the fixed effects fairness domain (compensation versus punishment), interaction partner (baseline versus fairness partner) and their interactions, and participant as random intercept. Note that this analysis deviates from the set of preregistered analyses. Results showed a significant interaction between the interaction partner and fairness domain (*β* = −0.13, s.e. = 0.06, *z* = −2.14, *p* = 0.03). *Post hoc* inspections revealed that this effect is probably based on a higher mean frequency of prosocial decisions towards the fairness partner than the baseline partner in the compensation domain (fairness partner: 0.33; baseline partner: 0.30), 95% confidence intervals, however, overlap (fairness partner: [0.17, 0.52]; baseline partner: [0.19, 0.58]). This indicates that the difference between the fairness partner and baseline partner in the compensation domain may only be significant relative to the punishment domain (fairness partner: mean = 0.26; 95% CI = [0.15, 0.46]; baseline partner: mean = 0.26, 95% CI = [0.15, 0.45]). We next tested whether the type of incentive (for decisions in favour of the self versus for decisions in favour of the other) was successfully implemented for both fairness domains. In this vein, we conducted a logistic mixed model with a decision as the dependent variable, fairness domain, incentive type and their interaction as fixed effects, and participant as a random intercept. Results showed that the groups with an incentive in favour of the other decided more frequently in favour of the other than the groups with an incentive in favour of the self (main effect of incentive type: *β* = 2.20, s.e. = 0.52, *z* = 4.22, *p* < 0.001). The main effect of fairness domain and the incentive type × fairness domain interaction were not significant (*p*s > 0.35).

### 3.2. Confirmatory analyses

#### 3.2.1. Decision behaviour

**Figure 5.**
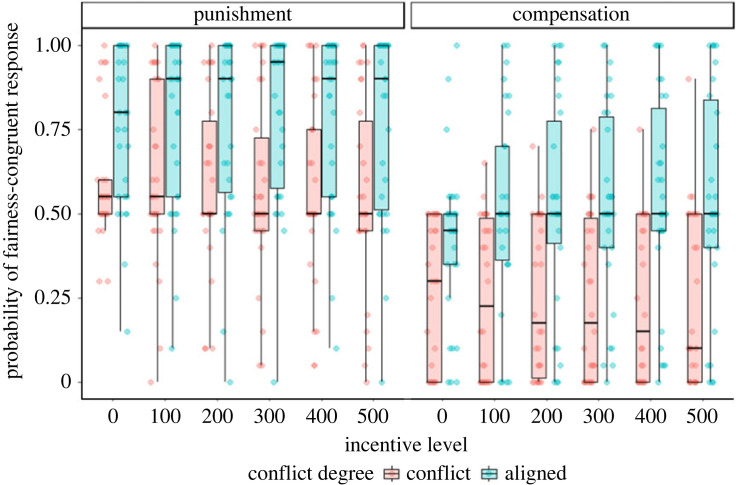
Effect of conflict degree and incentive level in the two fairness domains. Conflicting incentives diminish the frequency of fairness-congruent responses, i.e. decrease the frequency of decisions in favour of the other in the compensation domain and the frequency of decisions in favour of the self in the punishment domain. This effect is the larger, the higher the incentive level.

Using the model as specified in the study design table (table 2), we tested the hypotheses that were formulated in the theoretical section of this registered report. As predicted, conflicting monetary incentives diminished fairness-based behaviour (main effect of conflict degree: *β* = −2.01, s.e. = 0.50, *z* = −4.04, *p* < 0.001). Confirming H2, we observed an increasing effect of conflicting incentives with increasing incentive levels (incentive level × conflict degree interaction: *β* = −0.21, s.e. = 0.04, *z* = −4.95, *p* < 0.001). The effect of conflict was comparable between the compensation domain and the punishment domain, contradicting the assumption formulated in H3 (fairness domain × conflict degree interaction: *β* = 0.36, s.e. = 0.70, *z* = 0.52, *p* = 0.61, table 4 for full results and figure 5 for visualization).

**Table 4.** Results of the logistic mixed model with a response (congruent versus incongruent) as a dependent variable, conflict degree (conflicting versus aligned), fairness domain (punishment versus compensation), incentive level (0–500), the conflict degree × fairness domain interaction, and the conflict degree × incentive level interaction as fixed effects and participant as a random intercept. *N* = 14 400 observations and maximal variance inflation factor = 3.07. Significant fixed effects are highlighted in italics.

	*Β*	s.e.	*Z*	*p(z)*
(Intercept)	−0.018	0.345	−0.051	0.959
*conflict_degree*	*−2.014*	*0.498*	*−4.036*	*5.44 × 10^–5^*
*fairness_domain*	*2.181*	*0.497*	*4.384*	*1.14 × 10^–5^*
*incentive_level*	*0.138*	*0.031*	*4.479*	*7.50 × 10^–6^*
conflict_degree:fairness_domain	0.360	0.702	0.512	0.605
*conflict_degree:incentive_level*	*−0.209*	*0.042*	*−4.951*	*7.40 × 10^–7^*

### 3.3. Drift-diffusion modelling

We next tested our hypothesis with regard to the fairness-based decision process. Model comparison of the five estimated models (cf. table 2) based on the DIC values (table 5 for an overview of DIC values) showed that model V2, the model allowing the drift rate *v* to vary by conflict degree and fairness domain, best explained participants' behaviour. Hence, we used the parameter estimates of model V2 to test further hypotheses.

**Table 5.** Overview of the DIC values. The winning model (V2) is highlighted in italics.

	models	specification	DIC
null model	M0	—	10 960.95
influence on *v*-parameter models	V1	*v*∼*conflict degree*	10 966.71
	*V2*	*v*∼*conflict degree + fairness domain*	*10 947.07*
influence on *z*-parameter models	Z1	*z*∼*conflict degree*	10 962.80
	Z2	*z*∼*conflict degree* *+* *fairness domain*	10 961.20

The model comparison showed that conflicting incentives most strongly influence the decision process with regard to the speed of information accumulation, i.e. the *v*-parameter. Comparing the estimated *v*-parameters showed that the speed of information accumulation was higher in the two aligned groups than in the two conflict groups (99.5% probability of the parameters *v*_aligned_ being greater than *v*_conflict_ based on the posterior distributions, see section ‘Details of the drift-diffusion modelling’). This result lends support for H4, in which we hypothesized that conflicting incentives reduce the efficiency of choosing the fairness-congruent decision option. Moreover, for both the aligned as well as the conflict groups, the *v*-parameter was larger in the punishment domain as compared with the compensation domain.

Based on the model comparison results, the hypothesis that conflicting incentives reduce the initial bias towards the congruent decision option (H5) was not confirmed.

In H6, we assumed that the effect of conflicting incentives is different for the punishment and the compensation domain. In contrast to our prediction, there were no differential effects for the two fairness domains (39% probability for a larger effect in the punishment domain, i.e. 61% probability for a larger effect in the compensation domain).

### 3.4. Exploratory analyses

In addition to the preregistered analyses, we conducted two exploratory analyses in which we explored potential influences of relevant trait dimensions based on previous studies and tested a second model space of DDMs.

### 3.5. Influence of trait measures

In the first exploratory analysis, we tested whether trait measures were related to response behaviour in the different groups. Building on previous evidence for trait-dependent modulation of punishment and compensation behaviour in third-party paradigms, we explored the relationships between trait anger, altruism, and justice sensitivity and decision-making in our paradigm [87–90]. To do so, we correlated response behaviour in the four groups with the respective individuals' trait score on the anger scale [68,69], the altruism scale [71,72] and the three subscales of the justice sensitivity scale (JS_observer_, JS_victim_ and JS_perpetrator_; [70]). JS_observer_ scores capture how sensitive an individual reports to be to *observed* injustice. JS_victim_ and JS_perpetrator_ reflect the sensitivity to injustice experienced in the role of the victim and the perpetrator, respectively. In the conflict compensation group, results showed a positive correlation between fairness-congruent behaviour and the scores on the JS_perpetrator_ subscale (*p*_uncorrected_ = 0.001; *p*_Bonferroni_ = 0.005), indicating that the higher an individual's sensitivity to injustice in the role of the perpetrator, the more likely this individual was to show compensation despite monetary incentives to do the opposite. All other correlations did not reach significance on a corrected threshold (all *p*s_Bonferroni_ > 0.10)

### 3.6. Accounting for incentive levels in the drift-diffusion model

**Figure 6.**
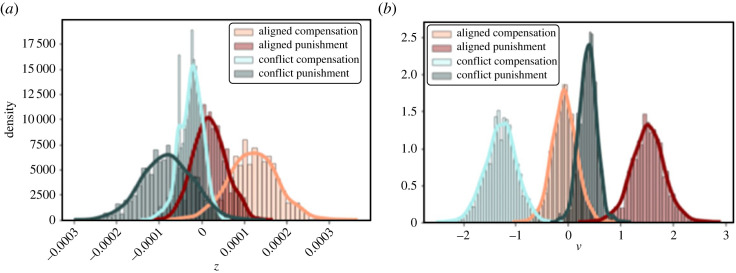
Results of the exploratory DDM analyses. (*a*) Posterior distribution of the regression weights indicating the influence of incentive level on the *z*-parameter. Less reach of the distribution beyond the 0-value corresponds to a larger effect. (*b*) Posterior distribution of the *v*-parameter. Less overlap between the orange and light blue (compensation domain), and the dark green and dark red (punishment domain) distribution, corresponds to a larger effect of conflicting incentives on the respective fairness-based behaviour.

In the second exploratory analyses, we tested a second model space of DDMs, in which we examined whether the trial-wise incentive level influences the initial bias (*z*-parameter) towards the fairness-congruent decision option. Results showed that for all groups except the conflict compensation group, a model accounting for a modulation of the *z*-parameter by incentive level better explained participants' behaviour than a model that does not (based on the DIC values). Comparing the posterior distributions for the effect weights showed that in the aligned compensation group, higher incentive levels were associated with a larger bias towards the fairness-congruent decision option (98.3% probability), and marginally with a smaller bias in the conflict punishment group (91.0%). The incentive level did not markedly influence the *z*-parameter in the conflict compensation group (81.1%) or the aligned punishment group (68.3%). Furthermore, these analyses replicated the confirmatory results that offering conflicting incentives decreases the *v*-parameter, i.e. the efficiency of the decision process (compensation: 99.8%, punishment: 100%, figure 6 for visualization).

## 4. Discussion

**Figure 7.**
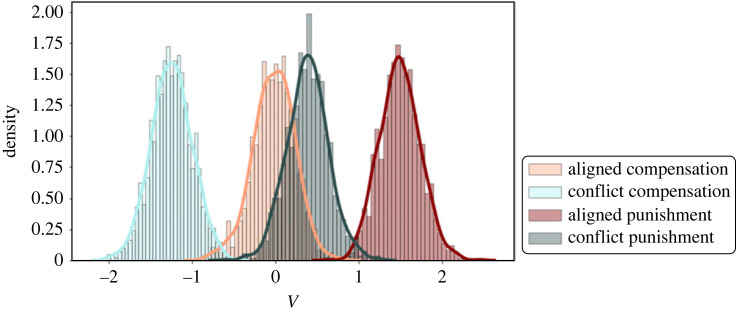
Posterior distributions of the estimated *v*-parameter in the four groups. Conflicting incentives (blue) markedly reduce the *v*-parameter as compared with aligned incentives (red). Generally, the decision process in favour of the self (punishment groups, darker shades) is more efficient than the decision process in favour of the other (compensation groups, lighter shades).

In this study, we investigated whether and how financial incentives influence the processing of fairness-congruent decisions in two different domains, i.e. fairness-based punishment (restoring fairness by punishing the norm violator) or fairness-based compensation (restoring fairness by compensating the victim of norm violation). First, our results showed a lower number of fairness-congruent decisions when conflicting monetary incentives were offered (figure 5). This effect was comparable across conditions. To specify the underlying mechanism, we used DDM, i.e. a method that allows for investigating different subcomponents of the decision process [46,75,76]. The results of the DDM analysis revealed that conflicting incentives were associated with a lower drift rate towards the fairness-congruent decision option (figure 7). Together, these results indicate that paying participants for making fairness-incongruent decisions diminishes the frequency and efficiency of fairness-congruent decision behaviour. In more detail, we show that offering financial incentives for behaving unfairly can undermine a person's willingness to compensate individuals that have suffered from unfair behaviour as well as the propensity to punish unfair behaviour.

In previous research, fairness-based punishment has been investigated with third-party punishment paradigms that give observers the option to invest their own points to punish norm-violators. Overall, the results showed a decline in the frequency of fairness-based punishment with increasing costs for the observer [22,27–33,91]. These results indicate that fairness-based punishment is modulated by punishment costs. Extending these previous findings, our results show that fairness-based punishment can be undermined by incentives that reward the acceptance of norm violations. Using an elegant modification of the third-party punishment paradigm, Rai [92] performed an online study in which participants had to perform a boring arithmetic task instead of giving up money to punish the norm-violator. Participants received a financial reward for performing this type of third-party punishment, corresponding to the set-up in the aligned punishment group in this study. Before the third-party punishment task, participants were asked to judge (Experiment 2) or to imagine (Experiment 3) moral transgressions, priming their moral concerns. Rai reported a reward-related decrease in punishment behaviour that was linked to moral concerns about taking rewards for punitive actions. Extending these results, our findings show that a monetary reward for unfair behaviour can have a similar undermining effect on fairness-based punishment as moral concerns. Moreover, we show that incentives can increase fairness-based punishment if punishing the norm-violator inherently increases the monetary outcome of the participant as in this study, and if moral concern is not explicitly primed.

Compared with previous work on fairness-based punishment, there are relatively few studies that addressed fairness-based compensation. Using costly compensation, previous studies have linked the extent of victim compensation to the extent of perceived fairness as well as personality traits such as empathy [25,39]. However, to the best of our knowledge, no previous study has investigated how compensation behaviour is influenced by continuously varying aligned versus conflicting incentives. Thus, this study extends previous results by showing that aligned financial incentives boost compensatory decision behaviour, whereas conflicting incentives can dampen compensation. Previous studies investigating whether participants preferred punishment or compensation behaviour, predominantly reported a preference for compensating the previously disadvantaged (e.g. [37,93,94]). In this study, however, we did not find indications for such a preference. Specifically, participants in this study more frequently chose the fairness-congruent decision option in the two punishment groups as compared with the two compensation groups. These results are in keeping with works which have observed that individuals tend to maximize their own outcome rather than compensate the disadvantaged (e.g. [42–44]). Moreover, the effect of conflicting financial incentives on fairness-based decision frequency was comparable across the two fairness domains. This finding, too, calls into question a general preference for compensation over punishment.

That said, previous studies have shown that observers who decided to compensate the disadvantaged were preferred over observers who had decided to punish the norm-violator [40,41]. Hence, in a live interaction paradigm, fairness-based compensation may be preferred and potentially be less easily overturned by conflicting monetary incentives than was the case in this study. Moreover, in these previous studies, participants could actively choose between punishment and compensation, while in our study the punishment and the compensation option were tested in a between-subject design to maximize the number of trials for DDM modelling.

In line with the present behavioural observations, the DDM results show that the drift rate parameter of the DDM, which is an indicator of decision efficiency [47,80], was generally larger in the two punishment groups as compared with the two compensation groups. This indicates general facilitation for fairness-based punishment compared with fairness-based compensation. Moreover, participants showed a higher drift rate for the incentivized decision, irrespective of whether this decision was fairness-congruent or fairness-incongruent. The observed increase in drift rate for the incentivized decision option is in line with previous studies showing a similar effect with regard to incentivized decisions in a binary dictator game [62], recognition memory [95] and numerosity discrimination [96]. In contrast to the observed increase in drift rate, the financial incentives did not affect the initial bias for fairness-congruent or fairness-incongruent decisions (captured by the *z*-parameter). Extending previous results, these findings show that offering financial incentives for fairness-congruent (aligned groups) or fairness-incongruent (conflict groups) behaviour affects the efficiency of the decision process, rather than participants' initial preferences to punish or compensate. That said, the results of exploratory DDM analyses suggest that, in addition to the drift rate, higher incentives might also shift the initial bias. This effect was most pronounced in the aligned compensation group which hints towards a stronger benefit of aligned financial incentives in the compensation domain. Future studies may hence specifically test whether this distinction also holds when other types of costs to punish or compensate are introduced.

In our study, a general tendency to maximize one's outcome was aligned with the fairness-based motivation to punish, as choosing in favour of the self towards a norm-violator increased one's own outcome. Likewise, a general tendency to maximize one's outcome was aligned with offering a financial incentive, as choosing in accordance with the incentive also increased one's own outcome. The finding that fairness-congruent behaviour correlated with justice insensitivity suggests that the observed behaviour is driven by the activated fairness norm and not merely by outcome maximization. To disentangle the effects of fairness-based behaviour and monetary incentives, future studies may use non-monetary punishment, for example asking participants to perform a time-consuming task (cf. [92]).

Moreover, the current results were observed in an anonymous setting of an online study, using the dictator game. This set-up allowed us to test the effects of financial incentives on fairness behaviour in a diverse sample. Future research should test the generalizability of our results in situations involving direct social interactions (both in the laboratory and in real life) and other types of decisions.

## Data Availability

The data that support the findings of this study and the analysis scripts are available at the Open Science Framework at https://osf.io/bgka8.

## References

[1] Brasini M, Vecchio SD, Gregni E, Casali C, Mira F, Capuano N, Questa V, Giacomantonio M, Mancini F. 2018 Fairness is a more effective interpersonal motive than care for sustaining prosocial behaviour. Psychology **09**, 1426-1443. (10.4236/psych.2018.96087)

[2] Camerer CF. 2011 Behavioral game theory: experiments in strategic interaction. Princeton, NJ: Princeton University Press.

[3] Fehr E, Fischbacher U. 2004 Social norms and human cooperation. Trends Cogn. Sci. **8**, 185-190. (10.1016/j.tics.2004.02.007) 15050515

[4] Fehr E, Schmidt KM. 1999 A theory of fairness, competition, and cooperation. Q. J. Econ. **114**, 817-868. (10.1162/003355399556151)

[5] Bolton GE, Ockenfels A. 2000 ERC: a theory of equity, reciprocity, and competition. Am. Econ. Rev. **90**, 166-193. (10.1257/aer.90.1.166)

[6] Deutsch M. 1975 Equity, equality, and need: what determines which value will be used as the basis of distributive justice? J. Soc. Issues **31**, 137-149. (10.1111/j.1540-4560.1975.tb01000.x)

[7] Barber IV BS, English W. 2019 The origin of wealth matters: equity norms trump equality norms in the ultimatum game with earned endowments. J. Econ. Behav. Organ. **158**, 33-43. (10.1016/j.jebo.2018.11.008)

[8] Cherry TL, Frykblom P, Shogren JF. 2002 Hardnose the dictator. Am. Econ. Rev. **92**, 1218-1221. (10.1257/00028280260344740)

[9] Franco-Watkins AM, Edwards BD, Acuff Jr RE. 2013 Effort and fairness in bargaining games. J. Behav. Decis. Mak. **26**, 79-90. (10.1002/bdm.762)

[10] Jakiela P. 2011 Social preferences and fairness norms as informal institutions: experimental evidence. Am. Econ. Rev. **101**, 509-513. (10.1257/aer.101.3.509)

[11] Forsythe R, Horowitz JL, Savin NE, Sefton M. 1994 Fairness in simple bargaining experiments. Games Econ. Behav. **6**, 347-369. (10.1006/game.1994.1021)

[12] Columbus S, Böhm R. 2021 Norm shifts under the strategy method. Judgm. Decis. Mak. **16**, 1267-1289.

[13] Kimbrough EO, Vostroknutov A. 2016 Norms make preferences social. J. Eur. Econ. Assoc. **14**, 608-638. (10.1111/jeea.12152)

[14] Krupka EL, Weber RA. 2013 Identifying social norms using coordination games: why does dictator game sharing vary? J. Eur. Econ. Assoc. **11**, 495-524. (10.1111/jeea.12006)

[15] Engel C. 2011 Dictator games: a meta study. Exp. Econ. **14**, 583-610. (10.1007/s10683-011-9283-7)

[16] Corradi-Dell'Acqua C, Civai C, Rumiati RI, Fink GR. 2013 Disentangling self- and fairness-related neural mechanisms involved in the ultimatum game: an fMRI study. Soc. Cognit. Affect. Neurosci. **8**, 424-431. (10.1093/scan/nss014) 22287263 PMC3624953

[17] Fehr E, Gächter S. 2002 Altruistic punishment in humans. Nature **415**, 137-140. (10.1038/415137a) 11805825

[18] FeldmanHall O, Sokol-Hessner P, Van Bavel JJ, Phelps EA. 2014 Fairness violations elicit greater punishment on behalf of another than for oneself. Nat. Commun. **5**, 1-6. (10.1038/ncomms6306) PMC4266485 25350814

[19] Guo X, Zheng L, Zhu L, Li J, Wang Q, Dienes Z, Yang Z. 2013 Increased neural responses to unfairness in a loss context. Neuroimage **77**, 246-253. (10.1016/j.neuroimage.2013.03.048) 23562770

[20] Windmann S, Hein G. 2018 Altruism from the perspective of the social neurosciences. E-Neuroforum **24**, A11-A18. (10.1515/nf-2017-a047)

[21] De Quervain DJF, Fischbacher U, Treyer V, Schellhammer M, Schnyder U, Buck A, Fehr E. 2004 The neural basis of altruistic punishment. Science **305**, 1254-1258. (10.1126/science.1100735) 15333831

[22] Jordan JJ, Hoffman M, Bloom P, Rand DG. 2016 Third-party punishment as a costly signal of trustworthiness. Nature **530**, 473-476. (10.1038/nature16981) 26911783

[23] Lo Gerfo E, Gallucci A, Morese R, Vergallito A, Ottone S, Ponzano F, Locatelli G, Bosco F, Romero Lauro LJ. 2019 The role of ventromedial prefrontal cortex and temporo-parietal junction in third-party punishment behavior. Neuroimage **200**, 501-510. (10.1016/j.neuroimage.2019.06.047) 31233906

[24] Heffner J, FeldmanHall O. 2019 Why we don't always punish: preferences for non-punitive responses to moral violations. Sci. Rep. **9**, 1-13. (10.1038/s41598-019-49680-2) 31519991 PMC6744396

[25] Urbanska K, McKeown S, Taylor LK. 2019 From injustice to action: the role of empathy and perceived fairness to address inequality via victim compensation. J. Exp. Soc. Psychol. **82**, 129-140. (10.1016/j.jesp.2019.01.010)

[26] Bicchieri C. 2005 The grammar of society: the nature and dynamics of social norms. Cambridge, UK: Cambridge University Press.

[27] Fehr E, Fischbacher U. 2004 Third-party punishment and social norms. Evol. Hum. Behav. **25**, 63-87. (10.1016/S1090-5138(04)00005-4)

[28] Anderson CM, Putterman L. 2006 Do non-strategic sanctions obey the law of demand? The demand for punishment in the voluntary contribution mechanism. Games Econ. Behav. **54**, 1-24. (10.1016/j.geb.2004.08.007)

[29] Egas M, Riedl A. 2008 The economics of altruistic punishment and the maintenance of cooperation. Proc. R. Soc. B **275**, 871-878. (10.1098/rspb.2007.1558) PMC2599936 18198144

[30] Fan L, Lu L, Liang J. 2012 *The demand of the third party punishment* (SSRN Scholarly Paper ID 2295732). Social Science Research Network. See https://papers.ssrn.com/abstract=2295732.

[31] Jordan JJ, McAuliffe K, Rand DG. 2016 The effects of endowment size and strategy method on third party punishment. Exp. Econ. **19**, 741-763. (10.1007/s10683-015-9466-8)

[32] Molho C, Tybur JM, Van Lange PAM, Balliet D. 2020 Direct and indirect punishment of norm violations in daily life. Nat. Commun. **11**, 1. (10.1038/s41467-020-17286-2) 32647165 PMC7347610

[33] Nikiforakis N, Normann H-T. 2008 A comparative statics analysis of punishment in public-good experiments. Exp. Econ. **11**, 358-369. (10.1007/s10683-007-9171-3)

[34] Thielmann I, Spadaro G, Balliet D. 2020 Personality and prosocial behavior: a theoretical framework and meta-analysis. Psychol. Bull. **146**, 30-90. (10.1037/bul0000217) 31841013

[35] van Doorn J, Zeelenberg M, Breugelmans SM. 2018 An exploration of third parties' preference for compensation over punishment: six experimental demonstrations. Theory Dec. **85**, 333-351. (10.1007/s11238-018-9665-9) PMC6413721 30956365

[36] Crockett MJ, Kurth-Nelson Z, Siegel JZ, Dayan P, Dolan RJ. 2014 Harm to others outweighs harm to self in moral decision making. Proc. Natl Acad. Sci. USA **111**, 17 320-17 325. (10.1073/pnas.1408988111) PMC4260587 25404350

[37] Chavez AK, Bicchieri C. 2013 Third-party sanctioning and compensation behavior: findings from the ultimatum game. J. Econ. Psychol. **39**, 268-277. (10.1016/j.joep.2013.09.004)

[38] Hu Y, Scheele D, Becker B, Voos G, David B, Hurlemann R, Weber B. 2016 The effect of oxytocin on third-party altruistic decisions in unfair situations: an fMRI study. Sci. Rep. **6**, Article 1. (10.1038/srep20236) PMC4735743 26832991

[39] Leliveld MC, Dijk E, Beest I. 2012 Punishing and compensating others at your own expense: the role of empathic concern on reactions to distributive injustice. Eur. J. Soc. Psychol. **42**, 135-140. (10.1002/ejsp.872)

[40] Raihani NJ, Bshary R. 2015 Third-party punishers are rewarded, but third-party helpers even more so. Evolution **69**, 993-1003. (10.1111/evo.12637) 25756463

[41] Ozono H, Watabe M. 2012 Reputational benefit of punishment: comparison among the punisher, rewarder, and non-sanctioner. Lett. Evol. Behav. Sci. **3**, 2. (10.5178/lebs.2012.22)

[42] Gino F, Norton MI, Weber RA. 2016 Motivated Bayesians: feeling moral while acting egoistically. J. Econ. Perspect. **30**, 189-212. (10.1257/jep.30.3.189)

[43] Johansson LO, Gustafsson M, Olsson L, Gärling T. 2007 Weighing third-party fairness, efficiency, and self-interest in resource allocation decisions. J. Econ. Psychol. **28**, 53-68. (10.1016/j.joep.2006.01.007)

[44] Rodriguez-Lara I, Moreno-Garrido L. 2012 Self-interest and fairness: self-serving choices of justice principles. Exp. Econ. **15**, 158-175. (10.1007/s10683-011-9295-3)

[45] Forstmann BU, Ratcliff R, Wagenmakers E-J. 2016 Sequential sampling models in cognitive neuroscience: advantages, applications, and extensions. Annu. Rev. Psychol. **67**, 641-666, (10.1146/annurev-psych-122414-033645) 26393872 PMC5112760

[46] Ratcliff R, Smith PL, Brown SD, McKoon G. 2016 Diffusion decision model: current issues and history. Trends Cogn. Sci. **20**, 260-281. (10.1016/j.tics.2016.01.007) 26952739 PMC4928591

[47] Ratcliff R, McKoon G. 2008 The diffusion decision model: theory and data for two-choice decision tasks. Neural Comput. **20**, 873-922. (10.1162/neco.2008.12-06-420) 18085991 PMC2474742

[48] Bottemanne L, Dreher J-C. 2019 Vicarious rewards modulate the drift rate of evidence accumulation from the drift diffusion model. Front. Behav. Neurosci. **13**, 142. (10.3389/fnbeh.2019.00142) 31312125 PMC6614513

[49] Gesiarz F, Cahill D, Sharot T. 2019 Evidence accumulation is biased by motivation: a computational account. PLoS Comput. Biol. **15**, e1007089. (10.1371/journal.pcbi.1007089) 31246955 PMC6597032

[50] Chen F, Krajbich I. 2018 Biased sequential sampling underlies the effects of time pressure and delay in social decision making. Nat. Commun. **9**, 1-10. (10.1038/s41467-018-05994-9) 30177719 PMC6120923

[51] Saulin A, Horn U, Lotze M, Kaiser J, Hein G. 2022 The neural computation of human prosocial choices in complex motivational states. Neuroimage **247**, 118827. (10.1016/j.neuroimage.2021.118827) 34923133

[52] Yu H, Siegel JZ, Clithero JA, Crockett MJ. 2021 How peer influence shapes value computation in moral decision-making. Cognition **211**, 104641. (10.1016/j.cognition.2021.104641) 33740537 PMC8085736

[53] Toelch U, Panizza F, Heekeren HR. 2018 Norm compliance affects perceptual decisions through modulation of a starting point bias. R. Soc. Open Sci. **5**, 171268. (10.1098/rsos.171268) 29657747 PMC5882671

[54] Son J-Y, Bhandari A, FeldmanHall O. 2019 Crowdsourcing punishment: individuals reference group preferences to inform their own punitive decisions. Sci. Rep. **9**, 1. (10.1038/s41598-019-48050-2) 31406239 PMC6690944

[55] Puffer SM, Meindl JR. 1992 The congruence of motives and incentives in a voluntary organization. J. Organ. Behav. **13**, 425-434.

[56] Butz B, Harbring C. 2020 Donations as an incentive for cooperation in public good games. J. Behav. Exp. Econ. **85**, 101510. (10.1016/j.socec.2019.101510)

[57] Bonner SE, Sprinkle GB. 2002 The effects of monetary incentives on effort and task performance: theories, evidence, and a framework for research. Account. Organ. Soc. **27**, 303-345. (10.1016/S0361-3682(01)00052-6)

[58] Goldfeld K, Wujciak-Jens J. 2020 simstudy: illuminating research methods through data generation. J. Open Sour. Softw. **5**, 2763. (10.21105/joss.02763)

[59] RStudio Team. 2021 RStudio: integrated development environment for R. RStudio, PBC.

[60] Green P, MacLeod C. 2016 simr: an R package for power analysis of generalised linear mixed models by simulation. Methods Ecol. Evol. **7**, 493-498. (10.1111/2041-210X.12504)

[61] Hein G, Engelmann JB, Vollberg MC, Tobler PN. 2016 How learning shapes the empathic brain. Proc. Natl Acad. Sci. USA **113**, 80-85. (10.1073/pnas.1514539112) 26699464 PMC4711838

[62] Iotzov V, Saulin A, Kaiser J, Han S, Hein G. 2022 Financial incentives facilitate stronger neural computation of prosocial decisions in lower empathic adult females. Soc. Neurosci. **17**, 441-461. (10.1080/17470919.2022.2115550) 36064327

[63] Iotzov V, Weiß M, Windmann S, Hein G. 2022 Valence framing induces cognitive bias. Curr. Psychol. (10.1007/s12144-022-03797-2)

[64] Henrich J et al. 2006 Costly punishment across human societies. Science **312**, 1767-1770. (10.1126/science.1127333) 16794075

[65] Lutz J. 2016 The validity of crowdsourcing data in studying anger and aggressive behavior: a comparison of online and laboratory data. Soc. Psychol. **47**, 38-51. (10.1027/1864-9335/a000256)

[66] Engelmann JB, Meyer F, Ruff CC, Fehr E. 2019 The neural circuitry of affect-induced distortions of trust. Sci. Adv. **5**, eaau3413. (10.1126/sciadv.aau3413) 30891491 PMC6415955

[67] Grech PD, Nax HH. 2020 Rational altruism? On preference estimation and dictator game experiments. Games Econ. Behav. **119**, 309-338. (10.1016/j.geb.2019.10.004)

[68] Schwenkmezger P, Hodapp V. 1991 The state-trait anger expression inventory. Z. Klin. Psychol. Psychopathol. Psychother. **39**, 63-68. 2058240

[69] Spielberger CD. 1988 Manual for the state-trait anger expression inventory. Odessa, FL: Psychological Assessment Resources.

[70] Baumert A, Beierlein C, Schmitt M, Kemper CJ, Kovaleva A, Liebig S, Rammstedt B. 2014 Measuring four perspectives of justice sensitivity with two items each. J. Pers. Assess. **96**, 380-390. (10.1080/00223891.2013.836526) 24066854

[71] Carlo G, Randall BA. 2002 The development of a measure of prosocial behaviors for late adolescents. J. Youth Adolesc. **31**, 31-44. (10.1023/A:1014033032440)

[72] Rodrigues J, Ulrich N, Mussel P, Carlo G, Hewig J. 2017 Measuring prosocial tendencies in Germany: sources of validity and reliablity of the revised prosocial tendency measure. Front. Psychol. **8**, 2119. (10.3389/fpsyg.2017.02119) 29270144 PMC5723663

[73] Hein G, Morishima Y, Leiberg S, Sul S, Fehr E. 2016 The brain's functional network architecture reveals human motives. Science **351**, 1074-1078. 26941317 10.1126/science.aac7992

[74] Bates D, Mächler M, Bolker B, Walker S. 2015 Fitting linear mixed-effects models using lme4. J. Stat. Softw. **67**, 1-48. (10.18637/jss.v067.i01)

[75] Wiecki TV, Sofer I, Frank MJ. 2013 HDDM: hierarchical Bayesian estimation of the drift-diffusion model in python. Front. Neuroinformatics **7**, 14. (10.3389/fninf.2013.00014) PMC3731670 23935581

[76] Vandekerckhove J, Tuerlinckx F, Lee MD. 2011 Hierarchical diffusion models for two-choice response times. Psychol. Methods **16**, 44-62. (10.1037/a0021765) 21299302

[77] Nunez MD, Vandekerckhove J, Srinivasan R. 2017 How attention influences perceptual decision making: single-trial EEG correlates of drift-diffusion model parameters. J. Math. Psychol. **76**, 117-130. (10.1016/j.jmp.2016.03.003) 28435173 PMC5397902

[78] Servant M, Montagnini A, Burle B. 2014 Conflict tasks and the diffusion framework: insight in model constraints based on psychological laws. Cognit. Psychol. **72**, 162-195. (10.1016/j.cogpsych.2014.03.002) 24762975

[79] Wagenmakers EJ, Ratcliff R, Gomez P, McKoon G. 2008 A diffusion model account of criterion shifts in the lexical decision task. J. Mem. Lang. **58**, 140-159. (10.1016/j.jml.2007.04.006) 19122740 PMC2330283

[80] Voss A, Rothermund K, Voss J. 2004 Interpreting the parameters of the diffusion model: an empirical validation. Mem. Cognit. **32**, 1206-1220. (10.3758/BF03196893) 15813501

[81] Watanabe S. 2010 Asymptotic equivalence of Bayes cross validation and widely applicable information criterion in singular learning theory. J. Mach. Learn. Res. **11**, 3571-3594.

[82] Kraemer PM, Fontanesi L, Spektor MS, Gluth S. 2021 Response time models separate single- and dual-process accounts of memory-based decisions. Psychon. Bull. Rev. **28**, 304-323. (10.3758/s13423-020-01794-9) 32989719 PMC7870645

[83] Thomas AW, Molter F, Krajbich I, Heekeren HR, Mohr PNC. 2019 Gaze bias differences capture individual choice behaviour. Nat. Hum. Behav. **3**, Article 6. (10.1038/s41562-019-0584-8) 30988476

[84] Evans NJ. 2019 Assessing the practical differences between model selection methods in inferences about choice response time tasks. Psychon. Bull. Rev. **26**, 1070-1098. (10.3758/s13423-018-01563-9) 30783896 PMC6710222

[85] Spiegelhalter DJ, Best NG, Carlin BP, van der Linde A. 2002 Bayesian measures of model complexity and fit. J. R. Stat. Soc. **64**, 583-639. (10.1111/1467-9868.00353)

[86] Gelman A, Rubin DB. 1992 Inference from iterative simulation using multiple sequences. Stat. Sci. **7**, 457-472. (10.1214/ss/1177011136)

[87] Lotz S, Baumert A, Schlösser T, Gresser F, Fetchenhauer D. 2011 Individual differences in third-party interventions: how justice sensitivity shapes altruistic punishment. Negotiation Confl. Manag. Res. **4**, 297-313. (10.1111/j.1750-4716.2011.00084.x)

[88] Rodrigues J, Nagowski N, Mussel P, Hewig J. 2018 Altruistic punishment is connected to trait anger, not trait altruism, if compensation is available. Heliyon **4**, 962. (10.1016/j.heliyon.2018.e00962) PMC6262784 30533543

[89] Rodrigues J, Liesner M, Reutter M, Mussel P, Hewig J. 2020 It's costly punishment, not altruistic: low midfrontal theta and state anger predict punishment. Psychophysiology **57**, e13557. (10.1111/psyp.13557) 32108363

[90] Rodrigues J, Weiß M, Mussel P, Hewig J. 2022 On second thought … the influence of a second stage in the ultimatum game on decision behavior, electro-cortical correlates and their trait interrelation. Psychophysiology **59**, e14023. (10.1111/psyp.14023) 35174881

[91] Cheng X, Zheng L, Liu Z, Ling X, Wang X, Ouyang H, Chen X, Huang D, Guo X. 2022 Punishment cost affects third-parties' behavioral and neural responses to unfairness. Int. J. Psychophysiol. **177**, 27-33. (10.1016/j.ijpsycho.2022.04.003) 35405147

[92] Rai TS. 2022 Material benefits crowd out moralistic punishment. Psychol. Sci. **33**, 789-797. (10.1177/09567976211054786) 35486472

[93] Dhaliwal NA, Patil I, Cushman F. 2021 Reputational and cooperative benefits of third-party compensation. Organ. Behav. Hum. Dec. Process. **164**, 27-51. (10.1016/j.obhdp.2021.01.003)

[94] Lotz S, Okimoto TG, Schlösser T, Fetchenhauer D. 2011 Punitive versus compensatory reactions to injustice: emotional antecedents to third-party interventions. J. Exp. Soc. Psychol. **47**, 477-480. (10.1016/j.jesp.2010.10.004)

[95] da Silva-Castanheira K, Lalla A, Otto AR, Sheldon S. 2020 Modelling the effect of monetary incentives on recognition memory. CogSci.

[96] Dix A, Li S-C. 2020 Incentive motivation improves numerosity discrimination: insights from pupillometry combined with drift-diffusion modelling. Sci. Rep. **10**, 1. (10.1038/s41598-020-59415-3) 32054923 PMC7018719

